# Supplementary material for: Comparison of Predatory Performance among Three Ladybird Species, Harmonia axyridis, Coccinella septempunctata and Hippodamia variegata, Feeding on Goji Berry Psyllid, Bactericera gobica
Source: Insects. 2023 Dec 31;15(1):19. doi: 10.3390/insects15010019 (PMC10816942; doi:10.3390/insects15010019)
Supplement: Supplementary file 1 [file insects-15-00019-s001.zip › insects-2779639-supplementary.pdf]

Supporting information

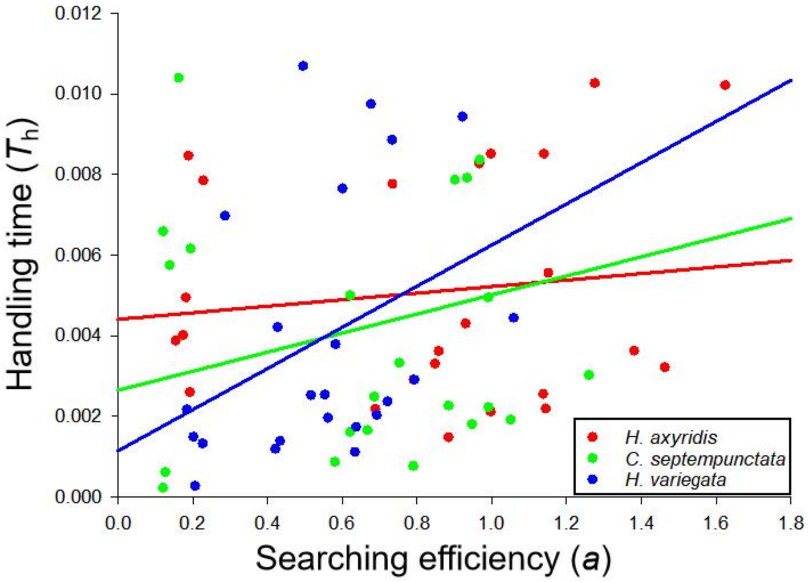

Figure S1 Relationship between the searching efficiency (*a*) and the handling time (T_h) for three ladybird species. Lines in various colors were each fitted using linear regression analysis. The parameters of all species treatments were positively correlated (Pearson's correlation coefficient r = 0.796, $P < 0.0001$, after reciprocal transformation for T_h), and within each species treatment (*H. axyridis*, $r = 0.869$; *C. septempunctata*, $r = 0.765$; *H. variegata*, $r = 0.855$).

Table S1. Summary for boxcox (*a*) ~ ladybird species * T_h via ANCOVA.

Response variable *a*	Sum Sq	*df*	Mean Sq	*F*	*P*	
Species	0.293	2	0.147	4.918	0.045	*
T_h	0.672	1	0.672	4.205	0.044	*
Species * T_h	0.112	2	0.056	9.349	0.037	*
Residuals	10.394	65	0.16			

Table S2. *P* value of factor ladybird species for searching efficiency (*a*) based on ANCOVA performed on pairs of ladybird species.

Pair of ladybird species	*P* (factor species)	*P* (interaction)
H. axyridis-C. septempunctata	0.796	0.721
H. axyridis-H. variegata	0.042*	0.038*
C. septempunctata-H. variegata	0.168	0.145

Table S3. Summary for boxcox (T_h) ~ ladybird species * *a* via ANCOVA.

Response variable T_h	Sum Sq	*df*	Mean Sq	*F*	*P*	
Species	46.158	2	23.079	3.176	0.031	*
a	113.585	1	113.585	5.786	0.019	*
Species * *a*	38.24	2	19.12	4.974	0.038	*
Residuals	1275.948	65	19.63			

Table S4. *P* value of factor ladybird species for handling time (T_h) via ANCOVA performed on pairs of ladybird species.

Pair of ladybird species	*P* (factor species)	*P* (interaction)
H. axyridis-*C. septempunctata*	0.731	0.816
H. axyridis-*H. variegata*	0.031*	0.023*
C. septempunctata-*H. variegata*	0.165	0.147

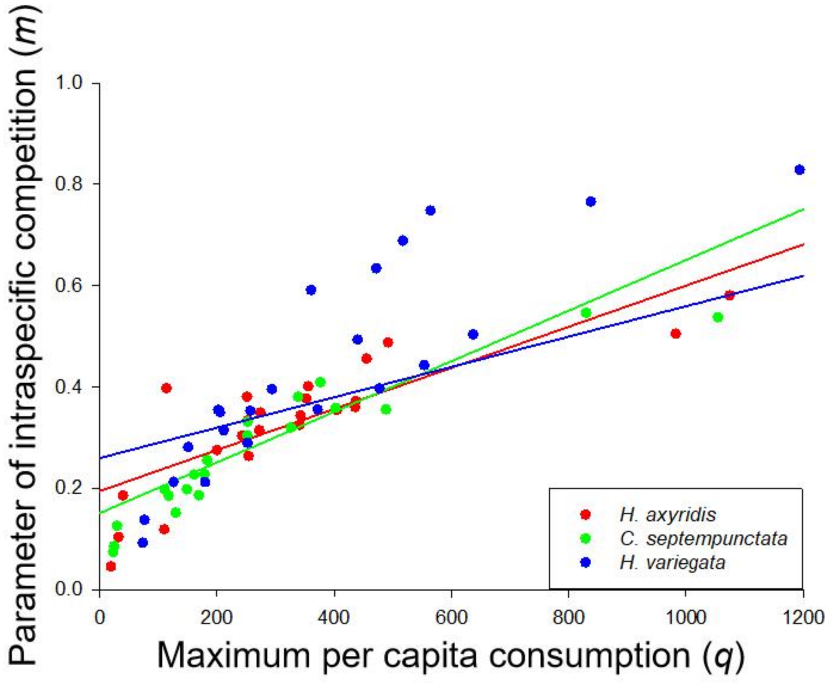

Figure S2 Relationship between the maximum consumption (*q*) and the intraspecific interaction parameter (*m*) for three ladybird species. Lines in various colors were each fitted using linear regression analysis. The parameters of all species treatments were positively correlated (Pearson's correlation coefficient $r = 0.815$, $P < 0.0001$, after log-transformation for *m* and reciprocal transformation for *q*), and within each species treatment (*H. axyridis*, $r = 0.820$; *C. septempunctata*, $r = 0.939$; *H. variegata*, $r = 0.764$).

Table S5. Summary for boxcox (*q*) ~ ladybird species * *m* via ANCOVA.

Response variable *q*	Sum Sq	*df*	Mean Sq	*F*	*P*	
Species	17927.18	2	8963.59	5.249	0.037	*
m	4558450.614	1	4558450.614	126.416	0	***
Species * *m*	136754.879	2	68377.44	11.896	0.015	*
Residuals	2199608.202	61	36059.151			

Table S6. *P* value of factor ladybird species for maximum consumption (*q*) based on ANCOVA performed on pairs of ladybird species.

Pair of species	*P* (factor species)	*P* (interaction)
H. axyridis-C. septempunctata	0.177	0.164
H. axyridis-H. variegata	0.422	0.348
C. septempunctata-H. variegata	0.01	0.014

Table S7. Summary for boxcox (*m*) ~ ladybird species * *q* via ANCOVA.

Response variable *m*	Sum Sq	*df*	Mean Sq	*F*	*P*	
Species	0.061	2	0.031	3.223	0.047	*
q	1.089	1	1.089	114.801	0	***
Species * *q*	0	2	0	3.02	0.048	*
Residuals	0.578	61	0.009			

Table S8. *P* value of factor ladybird species for intraspecific interaction parameter (*m*) based on ANCOVA performed on pairs of ladybird species.

Pair of ladybird species	*P* (factor species)	*P* (interaction)
H. axyridis-C. septempunctata	0.699	0.602
H. axyridis-H. variegata	0.077	0.086
C. septempunctata-H. variegata	0.012*	0.025*

Table S9. Maximum likelihood estimates (±SE) for parameters of the logistic model fit to the proportion of psyllids consumed versus the initial psyllid abundance. * Significant at $p < 0.05$; ** Significant at $p < 0.01$.

Stage	P_0	P_1	P_2	P_3
H. axyridis	2.135 **	−0.0621 **	3.6×10^{-4}	-8.8×10^{-7}
(SE)	0.296	0.0198	6.7×10^{-5}	2.2×10^{-7}
C. septempunctata	1.835 *	−0.0035 *	-3.1×10^{-5}	8.6×10^{-8}
(SE)	0.602	0.0277	7.6×10^{-6}	2.4×10^{-8}
H. variegata	−0.233	−0.0081 *	3.6×10^{-5}	-5.7×10^{-8}
(SE)	0.079	0.0055	8.2×10^{-6}	8.5×10^{-9}

Table S10. Parameters estimated from individual nonlinear regression for functional responses (Mean ± SE). For each parameter of a and T_h, differences between ladybird species were investigated using ANCOVA with the other parameter as independent variable and species as factor after Box–Cox transformation of the response variable and withdrawal of outlier. For one parameter, there was a significant interaction between the other parameter and species. We then focused on the comparison between pairs of ladybird species using the same approach.

Ladybird species	*H. axyridis*	*C. septempunctata*	*H. variegata*
a: searching efficiency	0.84±0.09 a	0.76±0.10 ab	0.55±0.05 b
T_h: handling time (min)	7.33±0.83 a	6.42±1.00 ab	5.67±0.97 b

Table S11. Parameters estimated from individual nonlinear regression for intraspecific interactions (Mean ± SE). For each parameter of q and m, differences between ladybird species were investigated using ANCOVA with the other parameter as independent variable and species as factor after Box–Cox transformation of the response variable and withdrawal of outliers. For one parameter, there was a significant interaction between the other parameter and species. We then focused on the comparison between pairs of ladybird species using the same approach.

Ladybird species	*H. axyridis*	*C. septempunctata*	*H. variegata*
q: maximum consumption	185.4±37.6 ab	203.35±41.3 b	133.4±26.9 a
m: intraspecific interaction parameter	0.33±0.03 ab	0.29±0.03 a	0.44±0.04 b
